# Comparison of Linear and Nonlinear Twist Extrusion Processes with Crystal Plasticity Finite Element Analysis

**DOI:** 10.3390/ma17051139

**Published:** 2024-02-29

**Authors:** Ülke Şimşek, Kemal Davut, Hiroyuki Miyamoto, Tuncay Yalçinkaya

**Affiliations:** 1 Roketsan Missiles Industries Inc., Ankara 06780, Türkiye; ulke.simsek@roketsan.com.tr; 2 Department of Aerospace Engineering, Middle East Technical University, Ankara 06800, Türkiye; 3 Department of Material Science and Engineering, Izmir Institute of Technology, İzmir 35430, Türkiye; kemaldavut@iyte.edu.tr; 4 Department of Mechanical Engineering, Doshisha University, Kyoto 610-0321, Japan; h.miyamoto@mail.doshisha.ac.jp

**Keywords:** crystal plasticity, linear twist extrusion, nonlinear twist extrusion, severe plastic deformation, texture analysis

## Abstract

The mechanical characteristics of polycrystalline metallic materials are influenced significantly by various microstructural parameters, one of which is the grain size. Specifically, the strength and the toughness of polycrystalline metals exhibit enhancement as the grain size is reduced. Applying severe plastic deformations (SPDs) has a noticeable result in obtaining metallic materials with ultrafine-grained (UFG) microstructure. SPD, executed through conventional shaping methods like extrusion, plays a pivotal role in the evolution of the texture, which is closely related to the plastic behavior and ductility. A number of SPD processes have been developed to generate ultrafine-grained materials, each having a different shear deformation mechanism. Among these methods, linear twist extrusion (LTE) presents a non-uniform and non-monotonic form of severe plastic deformation, leading to significant shifts in the microstructure. Prior research demonstrates the capability of the LTE process to yield consistent, weak textures in pre-textured copper. However, limitations in production efficiency and the uneven distribution of grain refinement have curbed the widespread use of LTE in industrial settings. This has facilitated the development of an improved novel method, that surpasses the traditional approach, known as the nonlinear twist extrusion procedure (NLTE). The NLTE method innovatively adjusts the channel design of the mold within the twist section to mitigate strain reversal and the rotational movement of the workpiece, both of which have been identified as shortcomings of twist extrusion. Accurate anticipation of texture changes in SPD processes is essential for mold design and process parameter optimization. The performance of the proposed extrusion technique should still be studied. In this context, here, a single crystal (SC) of copper in billet form, passing through both LTE and NLTE, is analyzed, employing a rate-dependent crystal plasticity finite element (CPFE) framework. CPFE simulations were performed for both LTE and NLTE of SC copper specimens having <100> or <111> directions parallel to the extrusion direction initially. The texture evolution as well as the cross-sectional distribution of the stress and strain is studied in detail, and the performance of both processes is compared.

## 1. Introduction

The size and uniform distribution of grains within polycrystalline metals play a pivotal role in their physical properties. In general, materials with smaller grain size exhibit increased strength and demonstrate enhanced properties at elevated temperatures compared to their coarser-grained counterparts (see, e.g., [1]). However, the presence of small grains can also generate certain problems such as a reduction in the ductility, and the material’s ability to withstand cyclic loads, especially in low-cycle fatigue, can be affected (see, e.g., [2,3]). The problem observed in the low-cycle regime is obviously linked to reduced ductility and a higher proportion of grain boundaries, which in turn leads to early initiation and propagation of cracks [4], and it is not observed in the high-cycle case. Microstructures with ultrafine grains (UFGs), characterized by an average grain size less than one micron (1 μm), are essential in polycrystalline metals. These small grains must be evenly dispersed throughout the material and possess a significant number of grain boundaries to exhibit advanced and distinctive properties [5]. In addition to classical techniques such as inert gas condensation, application of severe plastic deformation (SPD), where the material is subjected to substantial plastic strains, has been quite popular in recent years for obtaining nanostructured materials. SPD processing involves the application of considerable hydrostatic pressure to a bulk solid, allowing for an extremely high strain to be induced without causing significant alterations to the overall dimensions of the workpiece (see, e.g., [6]). The resulting densely packed nanomaterials created through SPD are referred to as advanced structural and functional materials, suitable for a variety of practical applications [7,8]. Over the last three decades, an increasing interest in producing ultrafine-grained (UFG) structures has led to the development of numerous severe plastic deformation (SPD) processes. Equal-channel angular pressing (ECAP) [9], high-pressure torsion (HPT) [10], accumulated roll bonding (ARB) [11], multi-directional forging (MDF) [12], and twist extrusion (TE) [13] are among the most extensively investigated SPD processes. These widely studied SPD methods offer both opportunities and challenges for producing UFG materials to advance engineering structures. Advanced materials, particularly those in the ultrafine-grained category, are used in various diverse fields including aerospace, automotive, biomaterials, chemical sensors, construction, electronics, metal forming, information storage, and optics [14,15,16]. Among the various severe plastic deformation processes that have been developed, extrusion-oriented methods stand out due to their ability to preserve material dimensions and minimize the production of waste materials [17].

ECAP is an established technique for producing bulk UFG metallic materials [18,19]; however, ECAP may introduce significant damage in the form of shear bands, cracking, and segmentation (see, e.g., [20]). Additionally, ECAP has certain limitations, including its inability to efficiently process materials with substantial length due to the instability introduced by the pressing punch [21,22]. Furthermore, challenges arise from the short service life of the die and the intricate nature of the process, which together limit the widespread applicability of ECAP [23,24]. The HPT process offers significant advantages, such as consistent deformation, low deformation resistance, minimal porosity, and the absence of contamination [1,4,25]. The incorporation of HPT into industrial applications faces specific limitations, primarily related to the size and shape of the samples subjected to the process [26]. Moreover, the intricacies associated with manipulating the process parameters and the inherent unpredictability when it comes to anticipating the resulting outcomes pose challenges in the effective utilization of HPT for engineering applications [27]. Additionally, HPT and ECAP are ill-suited for larger bulk materials that demand both high strength and toughness. To address this, the accumulated roll bonding (ARB) process was developed. However, challenges arise from the accumulation of total plastic strain within the materials during the process, exacerbated by the non-hydrostatic nature of the rolling process. As a result, edge cracks may appear in the sheets, especially as the number of cycles increases [28].

Multi-directional forging (MDF) boasts a simple die geometry, high efficiency, and repeatability [29,30]. Nonetheless, its grain refinement capability falls short of other SPD methods, and grain size reduction is uneven [31,32]. Twist extrusion (TE) was designed to alleviate the non-uniform particle size distribution in SPD processes [33]. Research indicates that the mold channel geometry prevents changes in the specimen’s form, allowing for multiple extrusions and the accumulation of severe plastic deformation [34]. However, recent studies revealed that TE also exhibits inhomogeneous distribution characteristics [35], with strain increasing from the center to the periphery [36,37]. Achieving a homogeneous grain distribution requires multiple passes [38,39]. Furthermore, during TE, rigid body rotation of the workpiece results in high local strain and punch forces [40,41,42]. In TE, a notable phenomenon is the significant grain refinement that occurs during the initial passes [43]. However, after the third or fourth pass, depending on the specific material and processing conditions, the effectiveness of grain refinement decreases considerably [44,45]. Eventually, the mean grain size stabilizes at the lowest achievable level for the given material and temperature.

In order to address the limitations of traditional twist extrusion molds with linear deformation geometry (LTE), the nonlinear twist extrusion (NLTE) process was introduced by one of the co-authors of this work, and certain initial experimental observations were made using pure magnesium [46]. Numerical analyses of NLTE demonstrate advantages in the punch force, deformation distribution, strain evolution, and homogeneous plastic strain distribution compared to LTE [47]. Understanding the texture development and changes in material properties during the process can aid in optimizing the process parameters, which is the main purpose of the current work. To enhance and optimize the NLTE process, a deeper understanding of the development of texture and alterations in material properties during the procedure is pivotal. This comprehension is essential for refining the process parameters, including mold geometry, pass numbers, frictional interactions, process speed, and backpressure values. Despite significant progress in investigating the kinetics and kinematics of SPD methods, research concerning the texture evolution characteristics of these processes remains relatively limited. However, the examination of the crystallographic texture evolution arising from the ECAP process has been undertaken (see, e.g., [48]). This indicates a growing interest in exploring the textural aspects of SPD methods to enhance their efficiency and applicability. These investigations have demonstrated good agreement between the predicted texture evolution and the experimental observations. Furthermore, these studies have underscored the influence of the friction coefficient between the mold and the sample on the texture evolution [49]. In contrast, the crystallographic analysis of the LTE process has not aligned well with experimental results, with suggestions pointing towards grain thinning as a possible factor contributing to this disparity [50]. Studies of texture evolution during the design of the simple shear extrusion (SSE) process, achieved by varying the TE die and process, showed that consistent results were achieved between predicted and experimental textures. Additionally, the effect of backpressure on the texture evolution of SSE processes was demonstrated using CPFE analysis [51]. In helical extrusion processes, a combination of FEM flow simulation and the crystal plasticity model has successfully predicted deformation textures, further highlighting the potential of these computational tools [52]. These studies collectively highlight the strides being made towards understanding and predicting texture evolution in various SPD processes, although challenges remain in certain cases.

In this context, the current work concentrates on the crystal plasticity analysis of the classical twist extrusion and the nonlinear twist extrusion processes in a comparative manner, focusing mainly on the texture evolution. In order to achieve this more clearly, a single crystal copper specimen is extruded and the results are discussed in detail, which has not been done previously. The recently proposed nonlinear approach is studied using two different initial orientations and the results are analyzed in comparison to the classical process. Previous research indicates that NLTE offers advantages in the punch force, deformation distribution, and strain evolution for grain refinement, resulting in improved mechanical properties of the extruded materials. Modeling the texture evolution provides insights into the deformation behavior and helps to optimize the process parameters for the best NLTE extrusion outcome.

The paper is organized as follows. First, in Section 2 the finite element models of the extrusion processes are presented together with the constitutive framework. The calibration of the material parameters and homogenization process is also briefly discussed. Then, in Section 3 the results on the deformation behavior and the texture evolution are presented and discussed in a comparative manner. Finally, some concluding remarks are given in Section 4.

## 2. Extrusion Process Modeling and Constitutive Behavior

In this section, finite element models of LTE and NLTE processes are presented with a crystal plasticity formulation. Calibration of the material parameters using the homogenization approach is also briefly discussed.

### 2.1. Finite Element Models of NLTE and LTE Processes

**Figure 1:**
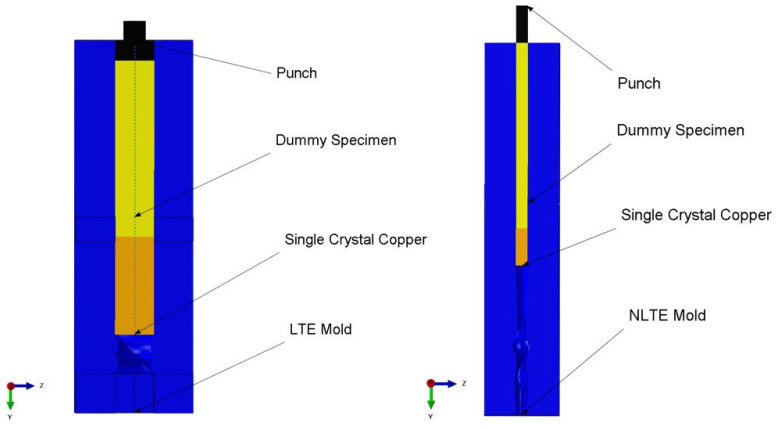
FE model of LTE (**left**) and NLTE (**right**) processes.

A finite element methodology is employed to model both the LTE and NLTE processes, which is the most common technique in the modeling of both classical and additive manufacturing processes (see, e.g., [53,54,55,56]). The finite element representation of LTE and NLTE is depicted in Figure 1. In the LTE procedure, a billet is forced through a die featuring two linear channels separated by a section with spiral geometry. As the billet is steadily rotated within the spiral section of the die, the cross-sectional shape of the billet, perpendicular to the extrusion axis, remains unchanged during its translational movement. The primary difference in the NLTE process is that the billet is extruded while retaining its rotation around the longitudinal axis. This feature prevents strain reversal and the rigid body rotation of the specimen.

In CPFE simulations for the LTE and NLTE processes, the mold model and punch are treated as rigid bodies. Encastre boundary conditions (u1 = u2 = u3 = R1 = R2 = R3 = 0) are applied to the rigid body reference point (center of mass) of the mold geometry and velocity boundary conditions (2 mm/s) are applied to the rigid body reference point of the punch geometry. Note that in the literature the punch speed in the twist extrusion process can vary between 2 mm/s and 8 mm/s (see, e.g., [57,58,59]). Friction is taken into account with a general contact algorithm between the mold and workpiece and the coefficient is approximately μ = 0.01, which might seem low [60,61]. There are various studies considering different values ranging from 0 to 0.15 in different SPD processes (see, e.g., [62,63,64]). In order to have a smooth comparative computational analysis the coefficient here is kept small considering numerical issues due to the nonlinear geometry in NLTE. In order to perform an experimental comparison, explicit analysis with a higher coefficient would be necessary. Nevertheless, the current study presents the deformation and texture evolution characteristics of these two different extrusion processes.

The models are built using individual SC copper samples, with square shapes for LTE and cylindrical shapes for NLTE. The rectangular prism with a square section has dimensions of 20 mm × 20 mm × 50 mm, while the cylindrical specimen has dimensions of 20 mm radius and 30 mm height. The mesh properties for the SPD models are provided in Table 1, while the computation duration is presented in Table 2. Note that application of the implicit crystal plasticity modeling technique is not entirely suitable for the presented extrusion analysis. However, since this is the initial attempt at the computational observation of texture evolution during the process it is acceptable. However, for further studies, other constitutive models or homogenization techniques at critical deformation paths could be employed considering the computational time.

### 2.2. Constitutive Models for NLTE and LTE Processes

**Figure 2:**
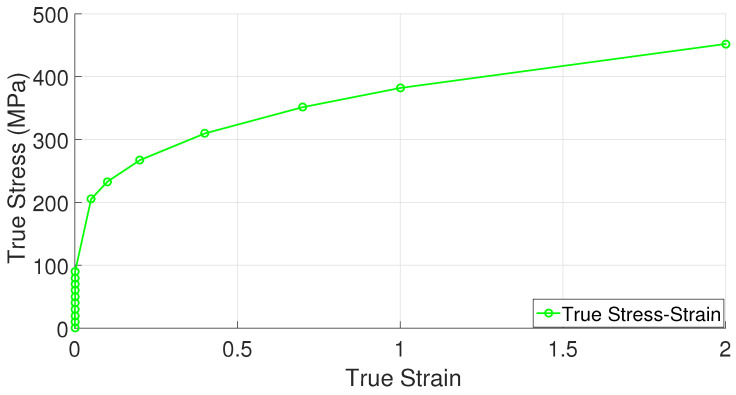
Stress–strain curve of polycrystalline copper material [47].

In the analysis, samples subject to shear deformation in the twist region are modeled with a rate-dependent crystal plasticity model. The part called dummy, which transmits the punch force to the sample to be examined throughout the process, is modeled with von Mises plasticity, using the stress–strain values of the polycrystalline copper material (Figure 2). The CPFE studies are based on a local UMAT (user-defined material model) subroutine based on the one developed by [65].

(1) $$\;F=F^{e}\cdot F^{p}$$

The deformation gradient **F** is decomposed multiplicatively into an elastic component $F^{e}$ and a plastic component $F^{P}$, as in Equation (1) (see, e.g., [66,67,68]); where $F^{p}$ denotes the stress-free intermediate configuration where the orientation of the lattice does not change and the plastic shearing occurs along well-defined slip planes. The symbol $F^{e}$ represents lattice stretching and rotation. It is assumed that the elastic properties remain unchanged in the presence of slip, meaning that stress is determined by $F^{e}$. The plastic velocity gradient, denoted as $L^{p}$, can be expressed in the following manner:

(2) $$L^{p}=\;{\dot{F}}^{p}\cdot {F^{p}}^{-1}=\sum_{\alpha }{\dot{\gamma }}^{\alpha }s^{\alpha }⊗m^{\alpha }$$

Here, the unit vectors $s^{\alpha }$ and $m^{\alpha }$ represent the slip direction and the nominal direction to the slip plane in the reference configuration, respectively, which can be determined for the current configuration as

(3) $$\;s^{*\alpha }=F^{e}s^{\alpha }$$

(4) $$\;m^{*\alpha }=m^{\alpha }{F^{e}}^{-1}$$

it is assumed that the crystalline slip follows Schmid’s law. The rate of slipping, denoted as ${\dot{\gamma }}^{\alpha }$, in a specific slip system α, depends exclusively on the Cauchy stress (σ) through the resolved shear stress ($\tau^{\alpha }$). The resolved shear stress for each slip direction can be found using Equation (5):

(5) $$\tau^{\alpha }=m^{*\alpha }\cdot det\left(F^{e}\right)\sigma \cdot s^{*\alpha }$$

According to Schmid’s law, the rate of slipping, ${\dot{\gamma }}^{\alpha }$, for the αth slip system in a rate-dependent crystalline solid is governed by the corresponding resolved shear stress $\tau^{\alpha }$, as expressed in Equation (6).

(6) $$\;{\dot{\gamma }}^{\alpha }={\dot{\gamma }}_{0}|\frac{\tau^{\alpha }}{g^{\alpha }}{|}^{n}sign(\tau^{\alpha })$$

The constant ${\dot{\gamma }}_{0}$ represents the reference strain rate for slip system α, while $g^{\alpha }$ is a variable that signifies the current strength of that particular system. Additionally, the rate sensitivity exponent is denoted by *n* (as in [69]). The evolution of the strength $g^{\alpha }$, which characterizes strain hardening, is described through the incremental relation shown in Equation (7).

(7) $$\;{\dot{g}}^{\alpha }=\sum_{\beta }h_{\alpha \beta }{\dot{\gamma }}^{\beta }$$

The term $h_{\alpha \beta }$ represents the slip hardening modulus, and the summation encompasses all activated slip systems. Specifically, $h_{\alpha \alpha }$ (with no summation) is referred to as the self-hardening modulus, while $h_{\alpha \beta }$ (for α≠β) is known as the latent hardening modulus. A straightforward expression for the self-hardening moduli can be employed, as presented in Equation (8) (as mentioned in [70]).

(8) $$\;h_{\alpha \alpha }=h\left(\gamma \right)=h_{0}sech^{2}|\frac{h_{0}\gamma }{\tau_{s}-\tau_{0}}|\left(no\;sum\;on\;\alpha \right)$$

Here, the parameter $h_{0}$ represents the initial hardening modulus, $\tau_{0}$ stands for the critical resolved shear stress, which is equal to the initial value of the current strength $g_{0}^{\alpha }$. Furthermore, $\tau_{s}$ denotes the stage I stress, often referred to as the break-through stress, at which significant plastic flow begins. Finally, γ represents the Taylor cumulative shear strain on all slip systems, as follows:

(9) $$\;\gamma =\sum_{\alpha }\int_{0}^{t}|{\dot{\gamma }}^{\alpha }|dt$$

The latent hardening moduli are determined by Equation (10).

(10) $$\;h_{\alpha \beta }=qh\left(\gamma \right)\left(\alpha \neq \beta \right)$$

The provided expressions for the hardening moduli, with the constant *q*, do not consider the Bauschinger effect in a crystalline solid.

### 2.3. Calibration of Crystal Plasticity Model

**Figure 3:**
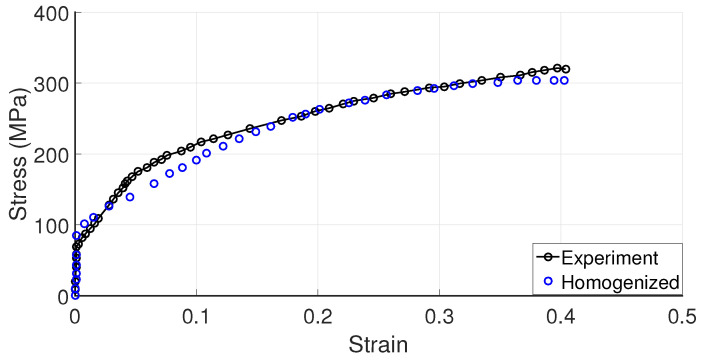
Homogenized stress–strain curve vs. experimental true stress–strain curve of polycrystal copper [47].

**Figure 4:**
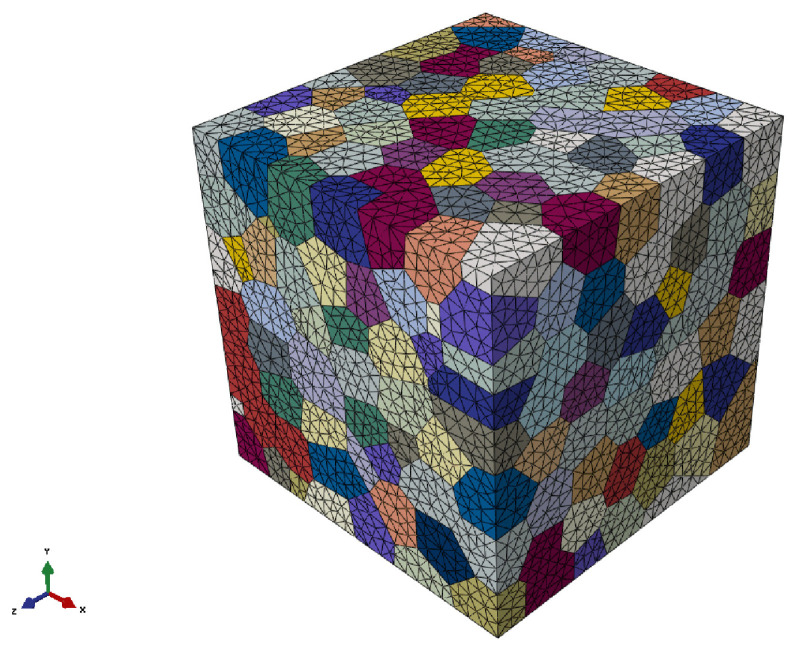
Representative volume element for polycrystalline copper with 500 grains.

**Figure 5:**
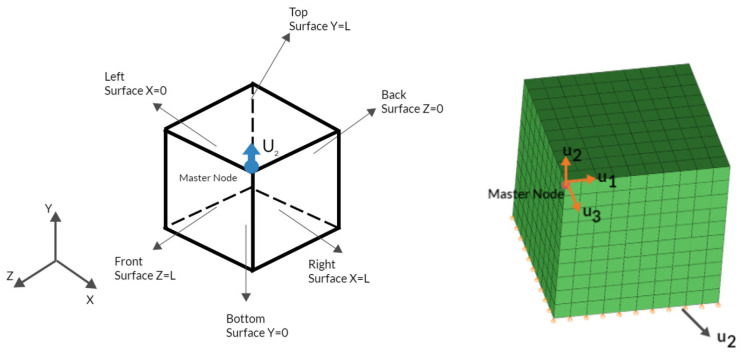
Boundary conditions of the representative volume element under axial loading to calibrate CPFE parameters.

In this section, the material parameters for single crystal (SC) copper are obtained following a homogenization scheme using polycrystalline material data. The experimental data for SC copper show variation in the literature and the parameters should be fitted for different initial orientation sets. Since the purpose is to illustrate qualitatively the performance of two different extrusion processes, such a comprehensive methodology is not followed here, and the stress-strain experimental data of polycrystal copper are utilized to calibrate the mechanical properties of SC copper (Figure 3). To accomplish this, the uniaxial tensile boundary conditions are enforced on a representative volume element (RVE) consisting of 500 grains with random orientations (Figure 4), ensuring polycrystalline isotropic behavior. The boundary conditions applied to the RVE under axial loading are depicted in Figure 5. For calibrating the parameters of the CPFE, homogenization procedures are employed and the obtained volume-averaged stress response is compared with the experimental tensile data. During the application of these boundary conditions, it is ensured that the stress triaxiality value ($T_{r}$), which is calculated by dividing the hydrostatic stress by the von Mises equivalent stress, remains constant at a ratio of 1/3 throughout the loading process and all the surfaces of the RVEs are kept straight. The following methodology can be applied to RVE for imposing different triaxiality values as well. Hydrostatic stress is calculated by

(11) $$P_{h}=\frac{\sigma_{1}+\sigma_{2}+\sigma_{3}}{3}$$

where $\sigma_{1}$, $\sigma_{2}$, and $\sigma_{3}$ are the principal stresses in the three principal directions. This naturally represents a case without shear deformation. Equivalent von Mises stress can be defined as

(12) $$\sigma_{v_{eq}}=\frac{1}{2}\sqrt{{\left(\sigma_{1}-\sigma_{2}\right)}^{2}+{\left(\sigma_{1}-\sigma_{3}\right)}^{2}+{\left(\sigma_{3}-\sigma_{2}\right)}^{2}}$$

And triaxiality can be found as

(13) $$T_{r}=\frac{P_{h}}{\sigma_{v_{eq}}}$$

To employ this boundary condition, firstly, the bottom surface of the RVE is constrained in the y–direction ($u_{2}$). The master node is selected at the corner of the RVE. The coordinate of this node should be ($L_{1},L_{2},L_{3}$) as it is located at the corner of the RVE, which enables us to couple the displacements of the surfaces and the master node. The unit cell’s edges are aligned with the coordinate axes, ensuring their straightness throughout deformation [71]. The displacements $u_{i}$, where i∈1,2,3, of all other nodes located on the surface containing node *M* are linked with the displacement $u_{i}$ of the node *M*. These couplings are established using the following linear Equations (14) [72,73,74].

(14) $$\begin{array}{c} \;u_{1}\left(L_{1},x_{2},x_{3}\right)-{\left(u_{1}\right)}^{M}=0,\\ \;u_{1}\left(0,x_{2},x_{3}\right)+{\left(u_{1}\right)}^{M}=0,\\ \;u_{3}\left(x_{1},x_{2},L_{3}\right)-{\left(u_{3}\right)}^{M}=0,\\ \;u_{3}\left(x_{1},x_{2},0\right)+{\left(u_{3}\right)}^{M}=0,\\ \;u_{3}\left(x_{1},L_{2},x_{3},\right)-{\left(u_{2}\right)}^{M}=0,\\ \;u_{2}\left(x_{1},0,x_{3}\right)=0. \end{array}$$

The overall responses of RVEs can be obtained through homogenization by taking the volume average:

(15) $$\bar{\sigma_{ij}}=\frac{1}{V}\int_{v}\sigma_{ij}dv\;with\;i,j=1,2,3$$

where $\bar{\sigma_{ij}}$ is the mesoscopic stress and $\sigma_{ij}$ is the microscopic Cauchy stress, and lastly, *V* is the volume of the RVE. As a result, $\bar{\sigma_{ij}}$ is determined for an RVE by summing the microscopic Cauchy stresses over each element with their associated integration points through the equation:

(16) $$\bar{\sigma_{ij}}=\frac{\sum_{m=1}^{N}{\left(\sum_{k=1}^{p}{\sigma_{ij}}^{k}v^{k}\right)}^{m}}{V}$$

where *N* is the number of elements, *p* is the total number of integration points, *v* is the integration volume, and *V* is the total volume. The material parameters of SC copper for the UMAT subroutine are illustrated in Table 3.

## 3. Results and Discussion

**Figure 6:**
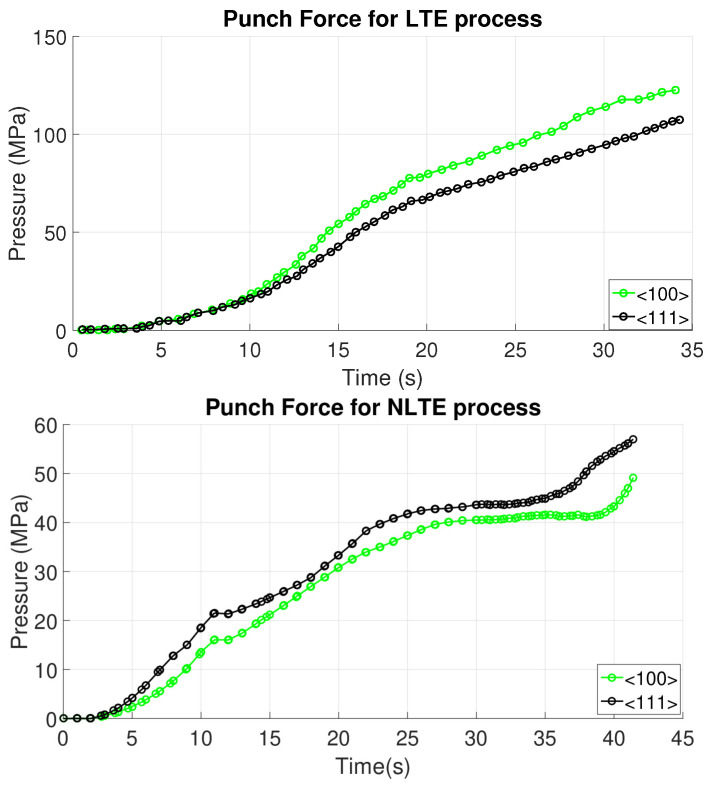
LTE and NLTE processes requires different punch forces based on the initial orientation of single-crystal copper.

In this study, CPFE simulations are conducted for both LTE and NLTE processes, using SC copper specimens, which are initially parallel to the <100> and <111> directions. During the simulations, an initial quadratic increase in the load is observed for both LTE and NLTE processes as the specimen head enters the twist zone. Following this abrupt rise, the gradient of the curve starts to decrease, and the punch force undergoes a slight elevation in both processes. This behavior has also been discussed in previous reports (see e.g., [35]). The necessary punch force is illustrated in Figure 6, showing that NLTE requires a lower punch force compared to LTE. This stands as a notable advantage of NLTE. The black curves represent the results for SC copper specimens oriented incipiently parallel to the <111> orientation, while the green lines represent SC copper specimens oriented initially parallel to the <100> orientation.

In the LTE process, an SC copper oriented initially in the <100> direction experiences a higher punch force compared to its <111>-oriented counterpart. Conversely, in the NLTE process, an SC copper oriented initially in the <111> direction requires a higher punch force throughout the process. In order to present a more accurate comparison, punch pressures should be evaluated considering the varying cross-sectional areas in both cases. However, the punch pressures are influenced considerably by the friction coefficient. In these calculations, a relatively low friction coefficient (approximately 0.01) is employed to address convergence issues related to implicit solvers in contact nonlinearities and frictional discontinuities. For a more realistic simulation with respect to real deformation processes, explicit analyses would indeed be better. However, despite their limitations, the implicit calculations serve as a reference for further explicit studies in these processes involving contact. Moreover, they allow a detailed comparison framework, which is the main purpose of the current study. An increase in the friction coefficient would result in higher punch forces for both processes. The abrupt increase in punch pressure in the LTE process is attributed to the sudden cross-sectional change, a characteristic not observed in the proposed NLTE process.

**Figure 7:**
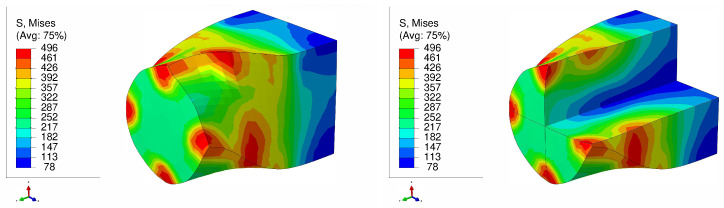
Von Mises stress distribution (MPa) of a single crystal of copper which is initially parallel to the <100> orientation during a single pass of LTE (**left**), with a cross-sectional view (**right**).

**Figure 8:**
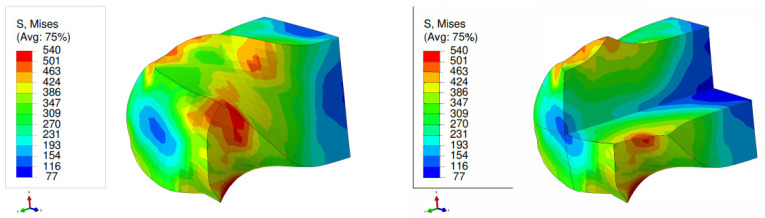
Von Mises stress distribution (MPa) of a single crystal of copper which is initially parallel to the <111> orientation during a single pass of LTE (**left**), with a cross-sectional view (**right**).

The von Mises stress distribution is depicted in Figure 7 for an SC copper initially parallel to the <100> orientation during the LTE process. The stress values exhibit a gradual rise from the center of the specimen towards its periphery, with the maximum stress levels concentrated at the surface edges. Conversely, in the case of a single crystal initially oriented in the <111> plane subjected to the LTE process, the stress evolution becomes more heterogeneous, leading to elevated stress values in peripheral regions (Figure 8).

**Figure 9:**
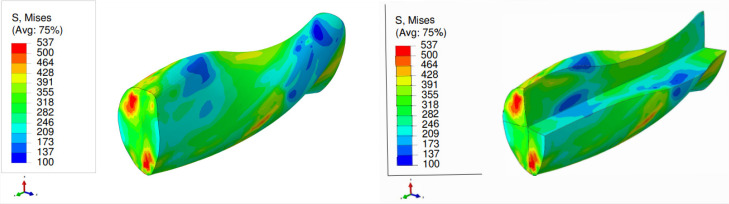
Von Mises stress distribution (in MPa) of a single crystal of copper which is initially parallel to the <100> orientation during a single pass of NLTE (**left**), with a cross-sectional view (**right**).

**Figure 10:**
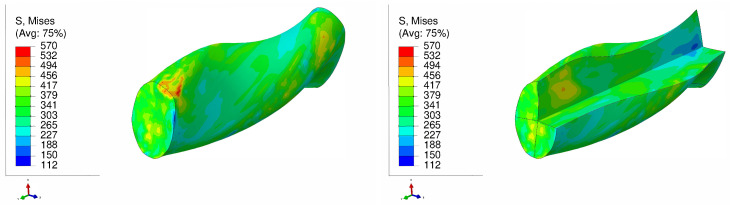
Von Mises stress distribution (MPa) of a single crystal of copper which is initially parallel to the <111> orientation during a single pass of NLTE (**left**), with a cross-sectional view (**right**).

Figure 9 and Figure 10 display the von Mises stress in the NLTE extrusion. Significantly, after the NLTE process stress becomes notably more uniform. High stress values are not only found in the peripheral region but also extend throughout the entire single crystal copper specimen, differing from the LTE process in the NLTE process. When comparing, SC copper specimens initially oriented in the <100> direction, they endure higher stress levels than their <111>-oriented counterparts.In both extrusion processes, the corners of the specimen bear the maximum stress levels. This is attributed to the fact that these regions come into contact with the interior surface of the die during the extrusion process.

**Figure 11:**
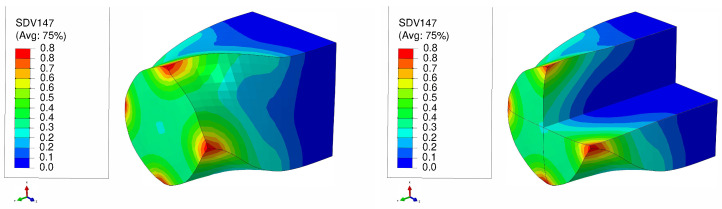
Plastic equivalent strain distribution of a single crystal of copper which is initially parallel to the <100> orientation during one pass of LTE process (**left**), with a cross-sectional view (**right**).

**Figure 12:**
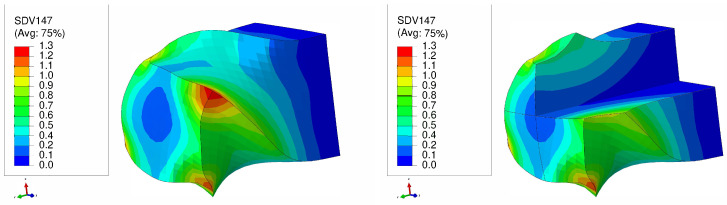
Plastic equivalent strain distribution of a single crystal of copper which is initially parallel to the <111> orientation during one pass of LTE process (**left**), with a cross-sectional view (**right**).

Figure 11 and Figure 12 present the distributions of equivalent plastic strain resulting from LTE processes for single copper crystals initially parallel to the <100> and <111> orientations, respectively. In LTE processes, the equivalent plastic strain exhibits lower magnitudes near the center of the specimen. From the center toward the surface of the specimen there is a gradual increase in equivalent plastic strain for both initial orientations. Notably, the highest strain accumulates at the corners, while the lowest strain is observed at the center. This distribution pattern is linked to the inhomogeneous strain distribution characteristic of the LTE process [41,50].

**Figure 13:**
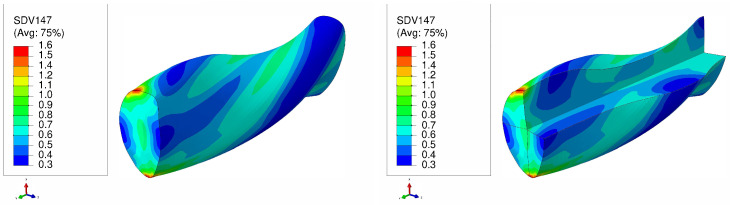
Plastic equivalent strain distribution of a single crystal of copper which is initially parallel to the <100> orientation during one pass of NLTE process (**left**), with a cross-sectional view (**right**).

**Figure 14:**
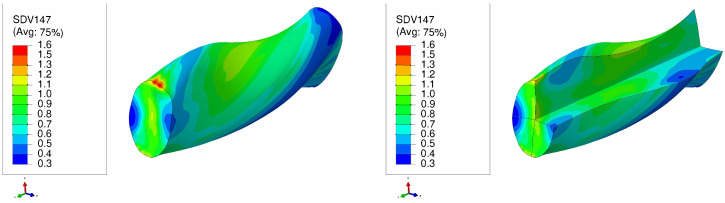
Plastic equivalent strain distribution of a single crystal of copper which is initially parallel to the <111> orientation during one pass of NLTE process (**left**), with a cross-sectional view (**right**).

Figure 13 and Figure 14 highlight that in NLTE processes the evolution of equivalent plastic strain is notably uniform. The distribution of equivalent plastic strain is more consistent from the center to the periphery of the specimen. Similar to LTE processes, initially directed <111>-orientation single crystal copper specimens experience greater plastic deformation compared to those initially directed to the <111> orientation for both processes.

Furthermore, during the NLTE process, the SC copper workpiece undergoes significantly higher plastic equivalent strain compared to the LTE process. This increased plastic deformation in NLTE processes is expected to enhance grain refinement, leading to further amplification of plastic strains.

**Figure 15:**
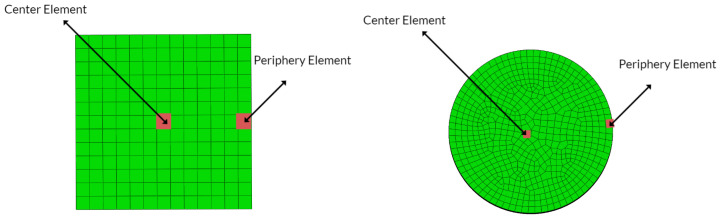
The analyzed elements, along with their local coordinates, are situated at the center and periphery for the LTE process (**left**) and the NLTE process (**right**).

To demonstrate the kinetic and the kinematic investigations of the LTE and NLTE processes, two representative elements from the workpieces have been chosen: one from the central section and another from the outer section of the specimen. These selected elements are visually emphasized in Figure 15.

Changes in reaction forces and boundary conditions over time are crucial factors in SPD processes. During the course of the process, loading directions, deformation modes, and the orientation of reaction forces on contact surfaces shift. Consequently, shear strain rates can vary due to these dynamic boundary conditions, and the anisotropic nature of a single crystal’s flow stress can also change under the influence of dynamic strain rates. While geometric factors tend to remain unaffected by strain rate changes, the motion of dislocations, interaction processes, and texture can exhibit increased sensitivity to strain rate fluctuations. These factors ultimately influence slip activity and microstructural evolution. Exploring plastic deformation behavior under such conditions can be challenging due to the difficulty of observing isolated dislocation groups and measuring microstructural evolution. In FCC metals, there are twelve distinct 111 slip systems that play a role in the plastic deformation process. However, the slip systems with identical Schmid factors do not always contribute equally to the deformation process. As dislocations form, aggregate, and arrange themselves into structures of lower energy, the internal stress state evolves. Local internal forces display heterogeneity and may either facilitate or impede dislocation glide on specific slip systems, regardless of their Schmid factors [75].

Rather than relying solely on Schmid factors and microstructural observations, anisotropy is best assessed through the concept of slip activity. Slip activity refers to the count and distribution of active slip systems [76,77]. These active slip systems are often indicated by dense slip lines and slip bands appearing on the most active planes. As strain rates increase, dislocation accumulation becomes more heterogeneous. Consequently, microstructures acquire a more directional character, resulting in the formation of slip bands on the most active planes. The increased heterogeneity amplifies changes in flow stress due to alterations in the loading direction, especially as strain rate increases [78,79].

**Figure 16:**
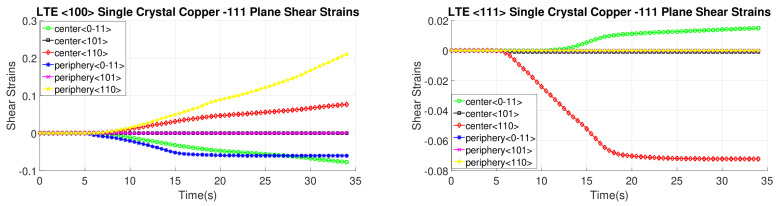
Shear strain evolution of -111 slip plane according to slip directions of <100>- and <111>-oriented single copper crystal.

**Figure 17:**
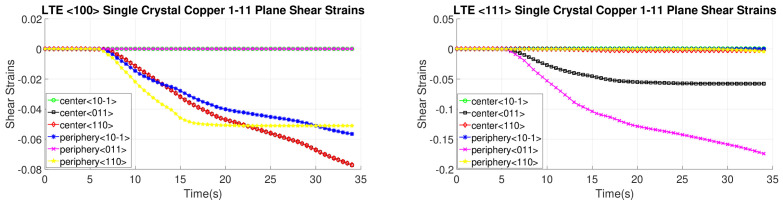
Shear strain evolution of 1-11 slip plane according to slip directions of <100>- and <111>-oriented single copper crystal.

**Figure 18:**
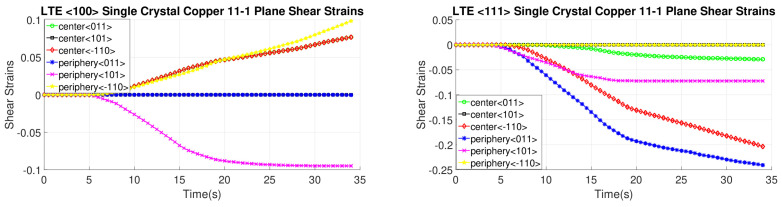
Shear strain evolution of 11-1 slip plane according to slip directions of <100>- and <111>-oriented single copper crystal.

**Figure 19:**
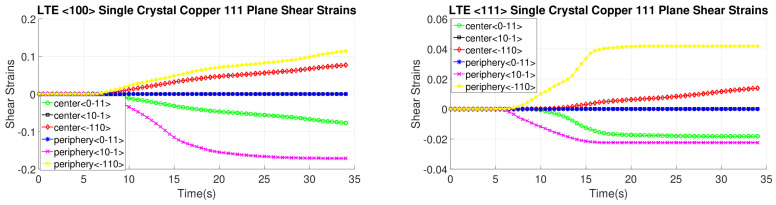
Shear strain evolution of 111 slip plane according to slip directions of <100>- and <111>-oriented single copper crystal.

Figure 16, Figure 17, Figure 18 and Figure 19 present shear strain variations over LTE process time, categorized by slip planes and the initial orientation of the SC crystals. These variations are analyzed for both the central and peripheral elements of the workpieces, focusing on the slip directions. Among the curves, the green, black, and red curves correspond to the center element, while the blue, pink, and yellow curves represent the peripheral element.

In the case of an SC copper initially parallel to the <100> orientation, the maximum shear strain values occur at the <-111> and <111> slip planes, along with the [110] and [10-1] directions, respectively, with a magnitude of 0.2. Conversely, for an initially <111>-oriented SC copper, the peak shear strain value is achieved on the <1-11> and <11-1> slip planes.

Notably, the most active shear plane and direction during the LTE process for the SC copper initially parallel to the <111> orientation in the peripheral element is the shear plane <11-1> with the [10-1] shear direction, as illustrated in Figure 18. In contrast, less activated slip directions are evident for the single copper crystal’s initially oriented to the <111> and <-111> slip planes, as depicted in Figure 16.

**Figure 20:**
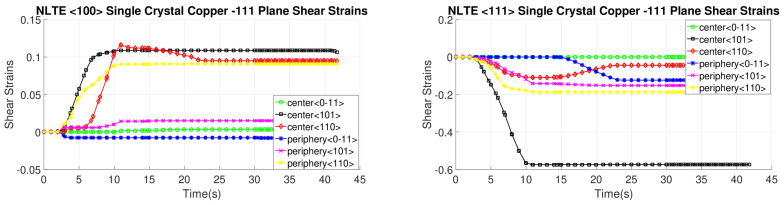
Shear strain evolutionof -111 slip plane according to slip directions of a single copper crystal initially directed to the <100> and <111> orientations.

**Figure 21:**
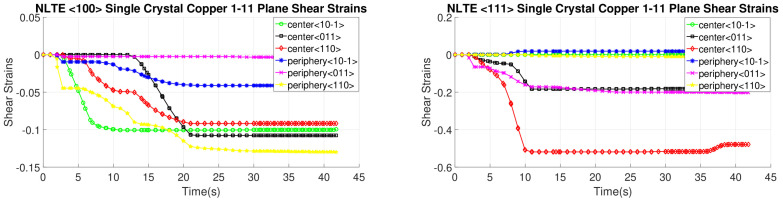
Shear strain evolution of 1-11 slip plane according to slip directions of single copper crystal initially directed to the <100> and <111> orientations.

**Figure 22:**
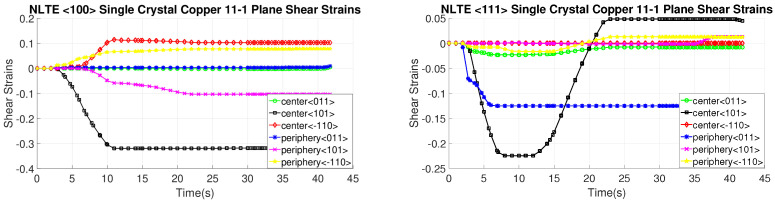
Shear strain evolution of 11-1 slip plane according to slip directions of single copper crystal initially directed to the <100> and <111> orientations.

**Figure 23:**
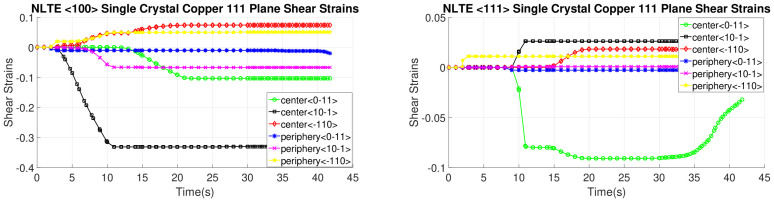
Shear strain evolution of 111 slip plane according to slip directions of single copper crystal initially directed to the <100> and <111> orientations.

Figure 20, Figure 21, Figure 22 and Figure 23 depict the evolution of shear strains over the processing time of the NLTE process, categorized by the shear plane, shear direction, and the initial orientations of a single copper crystal. These figures analyze both the central and peripheral elements of the workpieces, focusing on shear directions similar to the LTE process.

For a single copper crystal initially directed to the <100> orientation, the maximum shear strain values occurs at the <11-1> and <111> slip planes, accompanied by the [101] and [10-1] directions, respectively, with a shear strain magnitude of 0.3. In the case of an SC copper initially <111>-oriented, the maximum shear strain values occur on the <-111>, <1-11>, and <111> slip planes, accompanied by the [101], [110], and [101] directions, respectively.

These figures reveal that the most activated slip system belongs to the <-111> slip plane for the center element of an SC copper initially parallel to the <111> orientation, as indicated in Figure 20. Conversely, the less activated slip system is associated with the 11-1 slip plane for the center element of an SC copper initially parallel to the <111> orientation, as demonstrated in Figure 23. The NLTE process exhibits a more active shear system and higher shear strain values compared to the LTE process. Additionally, the phenomenon of strain reversal can be observed in the initially <111>-oriented single copper crystal’s <11-1> slip plane and [101] direction, as seen in Figure 22.

In FCC materials and their alloys, the stacking fault energy (SFE) holds a significant influence over the resulting crystallographic texture. The value of the SFE is contingent on the material type and is influenced by the presence and ratio of alloying elements. For instance, pure nickel (Ni) and aluminum (Al) possess very high SFEs, exceeding $4200\;{\mathrm{mJ}/m}^{2}$, while pure copper (Cu) exhibits an intermediate SFE, typically around 50 to 60 ${\mathrm{mJ}/m}^{2}$. The introduction of alloying elements frequently reduces the SFE to values below 20 ${\mathrm{mJ}/m}^{2}$. This characteristic of the SFE plays a crucial role in determining the primary deformation mechanism during plastic deformation. Specifically, materials with high to moderate SFE values tend to undergo deformation primarily through dislocation slip, while those with lower SFE values activate mechanisms such as twinning, leading to shear band formation. Both these mechanisms can significantly influence the deformation texture.

It is important to recognize that using ideal components in inverse pole figures (IPFs) and orientation distribution functions (ODFs) to describe texture evolution may be insufficient. Instead, textures can be represented as a spread of orientations, forming continuous orientation tubes across orientation space. The elemental textures of FCC metals, such as copper, brass, Goss, and S orientations, play a crucial role in comprehending the final texture of an SC of copper after deformation (see Table 4 for definitions). These texture components provide insights into the mechanical behaviors, physical and chemical properties, and overall performance of materials in engineering applications.

The control and understanding of texture evolution are crucial for the development of new materials [81]. In FCC materials featuring a high-to-medium SFE, grains typically experience rotation towards copper-type textures as deformation intensifies, and the primary deformation mechanism that prevails throughout the entire deformation process is dislocation slip. The SFE value plays a pivotal role in determining the primary mechanisms of plastic deformation. In metals characterized by a high SFE, like aluminum and copper, slip continues to be the dominant deformation mode, leading to the development of a rolling texture consisting of copper, brass, and S-texture components. In materials characterized by a low SFE, initially, copper-oriented grains tend to aggregate during the early stages of deformation. Nevertheless, as the loading process persists, these grains gradually undergo rotation toward brass-type textures, ultimately becoming the predominant texture after experiencing substantial deformations. The texture observed in low-SFE metals, characterized by a significant brass component and a minor Goss orientation, is commonly referred to as an alloy-type texture [82].

**Figure 24:**
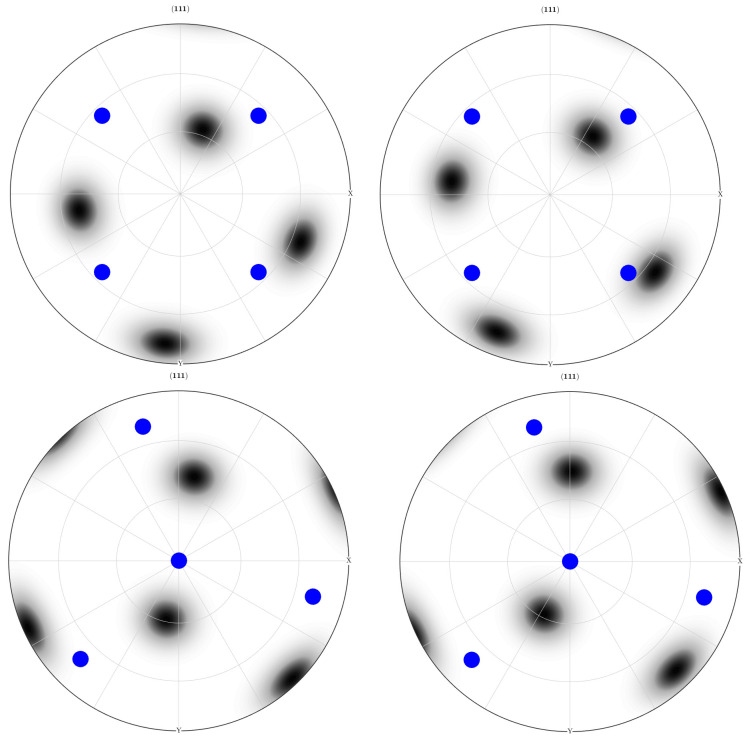
<111> Section pole figures of central elements (**left**) and peripheral elements (**right**) of a single crystal copper specimen after the LTE process. The blue points represent the initially directed <100> orientation (**top**) and <111> orientation (**bottom**).

**Figure 25:**
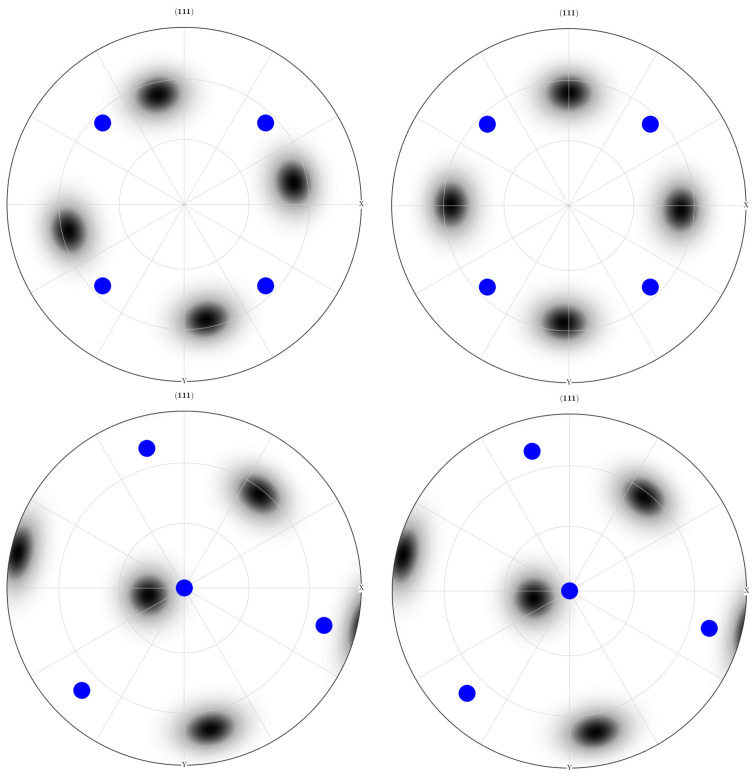
<111> Section pole figures of central elements (**left**) and peripheral elements (**right**) of a single-crystal copper specimen after the NLTE process. The blue points represent the initially directed <100> orientation (**top**) and <111> orientation (**bottom**).

After the initial pass of both the LTE and NLTE processes, the outcomes for the central and peripheral elements are depicted in Figure 24 and Figure 25, respectively. The initial orientation distributions exhibit significant variations after a single pass for each process. In the pole figures subsequent to the LTE process, the orientation distribution is reminiscent of the main orientation pole figures associated with copper and S-textures. Similarly, the central and peripheral elements of an SC copper specimen initially directed to the <100> orientation shows characteristics of the cube and S-textures after the NLTE process. Conversely, for SC copper workpieces initially directed to the <111> orientation, the central and peripheral elements exhibit orientation distributions resembling copper and S-textures after the NLTE process.

These pole figures indicate that both LTE and NLTE processes subject the elements of a single crystal copper specimen to shear deformation, with dislocation slip being the dominant deformation mechanism in both cases. Moreover, following a single pass, the orientation distributions for central and peripheral elements exhibit a greater alignment in the NLTE process compared to the LTE process. Upon comparing these results with the initial orientations, it becomes evident that both central and peripheral elements rotate about 45 degrees along the extrusion axis.

**Figure 26:**
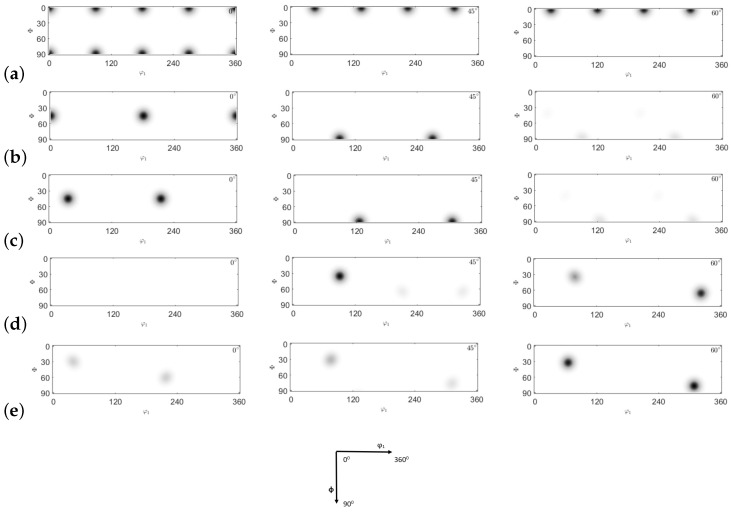
Comparison of ODF ($0^{\circ },45^{\circ },60^{\circ }$ section) obtained by texture components: (**a**) cube, (**b**) Goss, (**c**) brass, (**d**) copper, and (**e**) S-texture.

**Figure 27:**
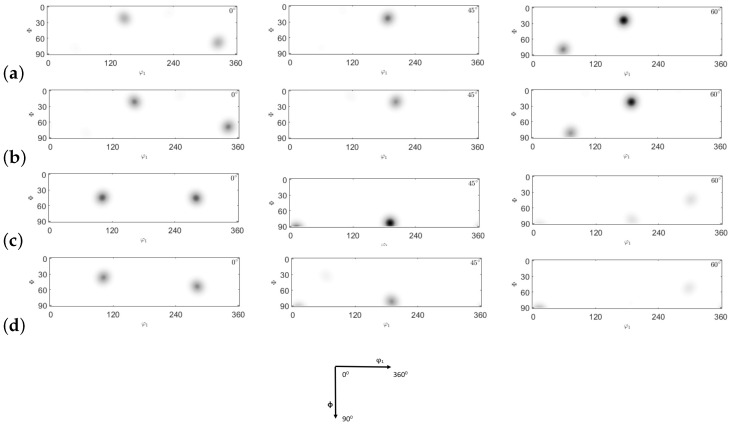
Comparison of ODFs ($0^{\circ },45^{\circ },60^{\circ }$ section) obtained by CPFE prediction of single crystal copper specimens: (**a**) central element initially oriented to <100>, (**b**) peripheral element initially oriented to <100>, (**c**) central element initially oriented to <111>, and (**d**) peripheral element initially oriented to <111> after LTE process.

**Figure 28:**
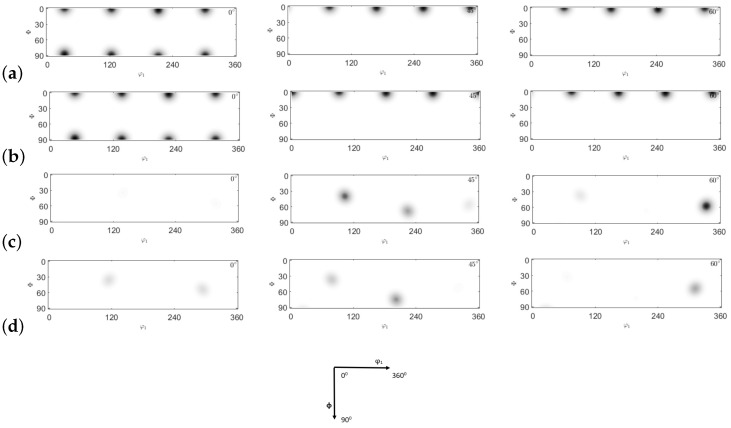
Comparison of ODFs ($0^{\circ },45^{\circ },60^{\circ }$ section) obtained by CPFE prediction of single crystal copper specimens: (**a**) central element initially oriented to <100>, (**b**) peripheral elements initially oriented to <100>, (**c**) central element initially oriented to <111>, and (**d**) peripheral element initially oriented to <111> after NLTE process.

ODFs are characterized by defining the X- and Y-directions as the transverse direction (TD) and the extrusion direction (ED), respectively. In this study, a copper-type sample symmetry is employed. Four primary texture components (cube, brass, S, and copper) are analyzed and presented in Figure 26. The texture evolution of the NLTE process is predicted and contrasted with that of the LTE process. Figure 27 and Figure 28 illustrate the ODFs of $\psi_{2}$ angles at $0^{\circ }$, $45^{\circ }$, and $60^{\circ }$ for the corresponding positions. Specifically, Figure 26a and Figure 26b display the initially <100>-oriented SC copper ODF results for central and peripheral elements, while Figure 26c and Figure 26d showcase the predicted ODF results for the initially <111>-oriented single copper crystal’s central and peripheral elements, respectively.

ODFs offer a valuable means of enhancing our comprehension of the texture evolution following the first pass of LTE and NLTE processes. Given that both LTE and NLTE processes are grounded in shear deformation, analyzing ODF sections with respect to the main textures can yield valuable insights. After a single pass of the LTE process, the ODFs for the central and peripheral elements exhibit discernible distinctions when compared to each other, regardless of whether the initial orientation is <100> or <111>.

Figure 27 presents a comparative view of the ODF results obtained from the LTE CPFE analysis of a single crystal copper specimen after a single pass. Comparing these ODF results with the primary texture components shown in Figure 27, in the case of an initially <100>-oriented SC copper specimen, both the central and peripheral elements display significant copper and S components. Conversely, for an initially <111>-oriented SC copper specimen, the copper and S component textures gradually weaken from the central to the peripheral elements, while the Goss and brass texture components become more effective.

Recent research has focused on understanding the influence of the crystallographic texture on fatigue resistance. Significant fatigue performance was observed in Goss grains of an Al–Cu–Mg alloy, whereas brass grains exhibited decreased resistance to fatigue crack propagation. The study demonstrated that a higher Goss/brass volume fraction ratio played a role in enhancing fracture toughness in the Al–Cu–Mg alloy [83].

The ODF CPFE results for SC copper specimens initially parallel to the <100> and <111> orientations are depicted in Figure 28. Upon comparing these results with the main texture components displayed in Figure 26, it becomes evident that the major components for both central and peripheral elements correspond to the cube texture components.

Evolution of the cube texture in FCC materials is a subject of special attention due to its crucial role in preferential growth during recrystallization heat treatments of cold-rolled sheets. This texture anisotropy contributes to the formation of defects during deep-drawing processes [84]. In the case of copper, the appearance of shear bands in the deformed structure provides alternative nuclei for recrystallization, exhibiting varying orientations with some inclination towards retained rolling texture components. These shear bands also disrupt the cube-oriented bands within the deformed structure, diminishing their favorability as nucleation sites. Consequently, the cube texture experiences significant weakening after recrystallization [85].

The propensity for shear banding is heavily reliant on the initial grain size of the material, thereby enabling the control of cube texture strength through this parameter. Fine initial grain sizes further refine the spacing of oriented bands, leading to a greater number of closely spaced cube bands. This not only facilitates the development of cube grains but also enhances the “orientation pinning” effect for other competing orientations, as exemplified in aluminum and discussed by Doherty et al. [86].

For initially <111>-oriented SC copper specimens, both central and peripheral elements exhibit strong S-textures following the NLTE process for a single pass. In metals with a high SFE, such as aluminum and copper, slip serves as the dominant deformation mode, and the rolling texture comprises copper, brass, and S-texture components with nearly equal intensities.

## 4. Conclusions

In this study, numerical simulations are employed to explore the extrusion process of individual single crystal copper workpieces, encompassing both the LTE and NLTE processes, while taking into account two distinct initial orientations of the workpieces. By adopting this methodology, a thorough and intricate comparison of the effectiveness of grain refinement, utilizing crystal plasticity finite element analysis, is conducted for these two severe plastic deformation techniques, marking the first instance of such an analysis in the literature.

The initial results of this investigation underscore the benefits of the NLTE process with respect to punch force and deformation distribution. While the LTE process leads to an increase in strain evolution from the central to the peripheral elements of the workpiece, the NLTE process experiences a more uniform distribution of plastic strain. Additionally, in shear deformation zones, the orientation distribution in the NLTE process appears to be more consistent compared to the LTE process. The NLTE process demonstrates a higher number of active shear systems and elevated shear strain values compared to the LTE process. Consequently, the workpiece undergoes more extensive plastic deformation during the NLTE process. Additionally, the NLTE process leads to a greater degree of grain refinement in the workpiece. Furthermore, the occurrence of strain reversal can be investigated specifically at the [11-1] slip plane and in the <101> direction. Moreover, the initial crystal orientation exerts an influence on the ultimate orientation outcomes of the processes. The NLTE process exhibits a higher number of active shear systems and higher levels of shear strain compared to the LTE process. Additionally, the NLTE process results in changes in crystal orientations through rotations along the extrusion direction.

The influence of backpressure and the friction coefficient on severe plastic deformation processes and microstructure is a well-established aspect. However, due to computational limitations associated with using the implicit UMAT code in this study, the consideration of the friction coefficient and backpressure load might have been insufficient. To mitigate this constraint, we plan to perform more comprehensive analyses employing an explicit methodology within the crystal plasticity framework. Subsequently, the acquired results will be subjected to further comparison with experimental observations. This next phase of our research will yield a more precise comprehension of how these parameters affect the simulation results.

## Figures and Tables

**Table 1:** Finite element model mesh properties of simulations of NLTE and LTE processes.

Design Element	Analysis Type	Element Type	Element Number
NLTE Mold	Implicit	Tetrahedral-C3D4	419316
NLTE Punch	Implicit	Hexahedral-C3D8R	1679
NLTE Specimen	Implicit	Hexahedral-C3D8R	48750
NLTE Dummy Specimen	Implicit	Hexahedral-C3D8R	15794
LTE Mold	Implicit	Tetrahedral-C3D4	33777765
LTE Punch	Implicit	Hexahedral-C3D8R	925
LTE Specimen	Implicit	Hexahedral-C3D8R	159989
LTE Dummy Specimen	Implicit	Hexahedral-C3D8R	36000

**Table 2:** Analysis times of LTE and NLTE processes.

Model	Punch Speed	CPU Time	Approximate Analysis Completion Time
UMAT CP LTE	2 mm/s	720 h:47 min:50 s	30 days
UMAT CP NLTE	2 mm/s	1078 h:17 min:21 s	45 days

**Table 3:** Single crystal copper UMAT subroutine material parameters.

Model	$C_{11}$	$C_{12}$	$C_{44}$	$\tau_{0}$	$\tau_{s}$	$h_{0}$	${\dot{\gamma }}_{0}$	n	q
UMAT CP	168,000 MPa	121,400 MPa	75,400 MPa	25 MPa	115 MPa	120 MPa	0.001 $s^{-1}$	17	1.4

**Table 4:** Elemental textures and pole figures for FCC materials relative to the [111], [110], and [100] planes [80].

Elemental Texture Name	Orientations	Bunge Euler Angles ($\varphi_{1}$ ϕ $\varphi_{2}$)	Pole Figures of Elemental Textures [111] [110] [100]
Cube	[001]<100>	(0 0 0)	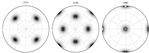
Goss	[011]<100>	(0 45 0)	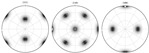
Brass	[0$\bar{1}$1]<211>	(35 45 0)	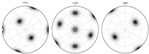
Copper	[$\bar{2}$11]<111>	(90 35 45)	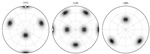
S	[12$\bar{3}$]<634>	(60 32 65)	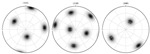

## Data Availability

Data are contained within the article.
